# Neuroimaging Biomarkers for Alzheimer’s Disease

**DOI:** 10.1186/s13024-019-0325-5

**Published:** 2019-06-07

**Authors:** Freddie Márquez, Michael A. Yassa

**Affiliations:** Department of Neurobiology and Behavior, Center for the Neurobiology of Learning and Memory, University of California Irvine, Irvine, CA 92697 USA

## Abstract

Currently, over five million Americans suffer with Alzheimer’s disease (AD). In the absence of a cure, this number could increase to 13.8 million by 2050. A critical goal of biomedical research is to establish indicators of AD during the preclinical stage (i.e. biomarkers) allowing for early diagnosis and intervention. Numerous advances have been made in developing biomarkers for AD using neuroimaging approaches. These approaches offer tremendous versatility in terms of targeting distinct age-related and pathophysiological mechanisms such as structural decline (e.g. volumetry, cortical thinning), functional decline (e.g. fMRI activity, network correlations), connectivity decline (e.g. diffusion anisotropy), and pathological aggregates (e.g. amyloid and tau PET). In this review, we survey the state of the literature on neuroimaging approaches to developing novel biomarkers for the amnestic form of AD, with an emphasis on combining approaches into multimodal biomarkers. We also discuss emerging methods including imaging epigenetics, neuroinflammation, and synaptic integrity using PET tracers. Finally, we review the complementary information that neuroimaging biomarkers provide, which highlights the potential utility of composite biomarkers as suitable outcome measures for proof-of-concept clinical trials with experimental therapeutics.

## Alzheimer’s disease and the need for biomarkers

Alzheimer’s disease (AD) is the most common cause for dementia [1]. Although there are various subtypes, the most common form is amnestic and severely impacts episodic memory [2]. With the exception of AD cases caused by genetic mutations (i.e. familial AD), age is the greatest risk factor. Currently, one in ten people 65 years of age or older have AD. In less than 60 years, life expectancy in the United States has increased by 9 years and the population of people 65 years of age and above has increased by 34 million people (16 million to 50 million). An estimated 5.5 million Americans currently suffer with AD and in the absence of effective treatment or a cure, this number could increase to 13.8 million by 2050 [1].

A critical goal of biomedical research is to establish indicators of AD during the preclinical stage (i.e. biomarkers) allowing for early diagnosis and intervention. These biomarkers are quantifiable characteristics of biological processes related to Alzheimer’s disease that are linked to clinical endpoints and thus can be used as surrogates for the disease process. Over the last decade, numerous advances have been made in developing biomarkers for AD using neuroimaging approaches. These approaches offer tremendous versatility in terms of understanding and targeting pathophysiological mechanisms such as structural decline (e.g. loss in volume, cortical thinning), functional decline (e.g. fMRI hyperactivity, altered network connectivity), white matter decline (e.g. diffusion anisotropy reduction, white matter pathology), and pathology aggregation (e.g. amyloid and tau PET).

In this review, we survey the state of the literature on neuroimaging approaches to developing novel biomarkers for AD, focusing on amnestic, late-onset (LOAD). We discuss advantages and limitations of each method and suggest that combining imaging modalities to create “composite biomarkers” may be a productive approach. These biomarkers may provide utility as potential outcomes for proof-of-concept clinical trials with experimental therapeutics.

## Pathology and spatiotemporal spread

Neuropathological staging criteria of AD-related changes originally indicated that although the distribution of beta-amyloid (Aβ) neuritic plaques varies widely, neurofibrillary tangles and neuropil threads show a distribution pattern that allow for the differentiation of six stages [3]. Stages I-II show alterations that are confined to the transentorhinal region, which spread to limbic (Stage III-IV), and finally to isocortical regions (Stage V-VI).

More recently, pathology studies have indicated that intraneuronal aggregations of the protein tau seem to precede the extracellular deposition of Aβ by approximately a decade [4, 5]. Notably, non-argyrophillic tau lesions are thought to first appear in the locus coeruleus prior to the appearance of argyrophillic tau lesions caused by neurofibrillary tangles (NFTs) within the transentorhinal region of the cerebral cortex [6]. Intraneuronal inclusions consisting of aggregated protein tau appear in selectively vulnerable cell types that appear to spread in a regionally and temporally specific manner that is independent of proximity to affected area [7].

A key advantage of using brain imaging techniques is that they operate at a higher level of spatiotemporal sensitivity than fluid biomarkers, thereby offering an opportunity to stage progression of the disease. Thus far, imaging using combinations of in vivo PET and MRI techniques have shown progression patterns that largely recapitulate staging based on post-mortem histology [8].

## Biomarker-based staging of preclinical Alzheimer’s disease

Identifying early biomarkers prior to the onset of disease symptoms is of critical importance to the field. It is thought that early intervention (i.e. during the pre-symptomatic stage) will be far more effective than later intervention, once the neurodegenerative cascade has set in. Historically, AD has been viewed as a disease of clinical symptoms in the clinical setting. By classifying AD in this manner, its diagnosis would likely include a considerable amount of non-AD cases as defined by its pathological characteristics. In 2011, the National Institute on Aging and the Alzheimer’s Association (NIA-AA) Working Group put forth staging criteria that incorporate neuroimaging biomarkers [9]. The authors presented a conceptual framework and operational research criteria for preclinical AD where Stage 1 is characterized by the presence of asymptomatic β-amyloidosis, or increased amyloid burden. Stage 2 includes neuronal injury and evidence of neurodegenerative change. Lastly, stage 3 additionally includes evidence of subtle cognitive decline, which is not yet sufficient for clinical diagnosis. The new research framework proposed by the NIA-AA defines AD pathologically with the use of biomarkers, which could potentially differentiate cases that clinically resemble AD such as hippocampal sclerosis. This framework additionally allows for staging using either fluid or neuroimaging biomarkers. However, certain features, which may be critical for the pathophysiology of the disease, could only be detected using imaging techniques. Hippocampal hyperactivity on task-activated functional MRI is one such example. Ewers et al. [10] and Leal and Yassa [11] include this feature in staging the disease and highlight that it seems to appear within a temporally constrained window.

Jack and Holtzman [12] proposed several time-dependent models of AD that take into consideration varying age of onset as well as co-morbid pathologies. Out of the five biomarkers proposed, three were imaging biomarkers (amyloid PET, structural MRI, and FDG PET). Importantly, anatomical information from imaging biomarkers provides crucial disease-staging information. This implies an advantage for imaging biomarkers over fluid biomarkers, because imaging can distinguish the different phases of the disease both temporally and anatomically.

The NIA-AA research framework has since been updated [13, 14] to focus on A/T/N criteria, first proposed by Jack and colleagues [15] and pave the path to more personalized diagnosis and treatment. The new framework highlights the value of positive amyloid biomarkers (A) to specifically indicate AD-related processes. Pathological tau (T) is only taken to indicate an AD-related process in the presence of amyloid positivity. Finally, (N) biomarkers are thought to provide nonspecific information about neuronal injury and neurodegenerative change.

The combination of amyloid with other biomarkers can then be used to stage AD progression. Additionally, according to this new framework, the presence of tau and neurodegeneration in the absence of amyloidosis is considered evidence for non-AD pathological processes. An important aspect of the 2018 NIA-AA working group framework is the flexibility to include additional biomarkers in future iterations. In our survey of neuroimaging methods, we will make the case that there are several methods for measuring A, T, and N pathologies, but also discuss new approaches to imaging additional biomarkers which may be integrated in biomarker models in the future (e.g. neuroinflammation).

## Imaging Amyloid Burden

Given the critical importance of identifying amyloid pathology in the brain as an early stage of AD progression, positron emission tomography (PET) scans with radiolabeled tracers specific to Aβ have become fairly commonplace in the research setting. The pathological Aβ peptide is generated by abnormal proteolytic processing of a physiological constituent of the nerve cell membrane, the amyloid precursor protein (APP). PET scans operate on the principle that positron-emitting radioligands accumulate in a region of interest. The positively-charged positrons encounter negatively-charged electrons, which results in annihilation releasing gamma photons that are detected by scintillation detectors [16]. This method can be used to image Aβ *in vivo* via radiolabeled tracers, which are injected via a bolus injection, followed by a waiting period to allow for uptake by brain tissue.

Amyloid tracers were developed via the modification of the histological dye, thioflavin-T, which has a high affinity to fibrillar and cerebrovascular amyloid, is cleared rapidly from normal brain tissue, and crosses the blood-brain barrier in sufficient amounts to be imaged *in vivo* [17]. Amyloid burden imaging was first explored with carbon-based tracers [^11^C] such as Pittsburgh Compound B (PiB), but the development of fluorine-based tracers [^18^F] has allowed for a wider availability of these longer lasting tracers facilitating widespread use. These tracers include florbetapir, florbetaben, and flutmetamol, which have an extended half-life (~110 minutes) as compared to [^11^C] tracers (20 minutes).

Most amyloid imaging studies point to the parietal cortices as the earliest sites of amyloid deposition [18]. Notably, these regions (posterior cingulate, restrosplenial cortex, precuneus) are heavily interconnected with the medial temporal lobes (MTL) [19], which are sites for early aggregation of tau pathology. Thus, the progression of the disease may be influenced by the anatomical and functional connectivity between the posterior cortices and the MTL. Amyloid tracers additionally bind to cerebrovascular amyloid. Cerebral Amyloid Angiopathy (CAA) is a feature of AD, is characterized by cortical vascular amyloid deposits, and is associated with cortical tissue loss, vascular dysfunction and cognitive decline [20, 21]. CAA severity is also associated with allocortical microinfarcts located in the hippocampal CA1 subfield [21]. Therefore, combining amyloid-PET with other imaging modalities may provide clues into the pathological sequence of events.

While amyloid tracers have produced similar qualitative findings across studies, institutions, and tracers, they vary in quantitative outcome measures of tracer retention. In an effort to standardize quantitative amyloid imaging measures, the Centiloid Project Working Group was formed in 2012 during the Alzheimer’s Imaging Consortium pre-meeting to the Alzheimer’s Association International Conference. The group works to harmonize across [^11^C] PiB and [^18^F] tracers using a percentile-based normalized system to address the variability of the method and tracer of each analysis (scales the outcome to a 0 to 100 scale). Despite these efforts, there still remain differences among tracers and in their sensitivity. Other factors that differ across studies include acquisition duration, target and reference region choice, partial volume correction, scanner differences, as well as differences in reconstruction algorithms and methods of attenuation correction.

A major limitation to amyloid imaging and studies of amyloid burden in general is a poorly understood relationship with cognition. It has been argued that since changes in amyloid may occur earlier than cognitive symptoms, such a relationship may not be expected. However, in the absence of a strong relationship, it remains unclear whether amyloid burden, in and of itself, is pathological or whether it is a sentinel for another pathology that may have more severe consequence on neural integrity. Understanding the latter is critical, especially as numerous clinical trials have targeted amyloid pathology as an attempt to modify the disease process.

Several studies have recently attempted to shed light on the relationship between amyloid and cognitive impairment [22–24]. These studies have found evidence for a link between Aβ accumulation and cognitive outcomes that appears to be mediated by neurodegenerative changes (e.g. cortical thinning and hippocampal volume loss). However, Aβ accumulation does not appear to be a precondition for neurodegenerative decline. For example, Wirth et al. [25] demonstrated that neurodegenerative changes could significantly predict cognitive performance in the absence of Aβ pathology. Most recently, it has been shown that subthreshold amyloid deposition predicts tau deposition in aging [26] suggesting that amyloid binding varies on a continuum. Despite its limitations, amyloid PET has been a tremendously informative tool in AD biomarker research, not only for staging disease progression, but additionally to select individuals for participation in biomarker-based clinical trials during the asymptomatic phase (secondary prevention trials), such as the A4 trial [27].

## Imaging Tau Burden

Tau is a neuronal protein that is produced throughout the nervous system and promotes self-assembly of axonal microtubules and stabilizes them [28]. Homeostatic shifts between a less highly phosphorylated state, where tau is bound to axonal microtubules, and a more highly phosphorylated state, where tau is soluble in the axoplasm, are enabled by axonal kinases and phosphates [29]. Changes in the equilibrium can give rise to conformational changes that lead to aggregation and changes in solubility that alters the functional role of tau and allowing for it to become resistant to autophagy and other mechanisms that regulate the removal of tau [30, 31].

Soluble hyperphosphorylated tau aggregates into spherical units of nucleation that then assembles linearly and forms ribbons of protofibrils with a β-sheet core. The absence or recurrence of twists enables classification of hyperphosphorylated tau into straight filaments, paired helical filaments (PHF) with regular twists, or irregularly twisted filaments. Recent evidence also suggests that tau pathology may spread trans-synaptically, in a prion-like fashion [32, 33] and that a critical component of the pathological cascade may be the conversion of tau monomers from an inert to a seed-competent form [34].

Development of selective tau PET tracers started as early as 2002 with quinolone and benzimidazole derivatives for their affinity to bind to PHFs. The cooccurrence of PHFs with Aβ provided an additional challenge as Aβ has the potential to also bind to the ligand, but a 25 fold selectivity for PHF over Aβ has been achieved [35]. Notably, the presence of other tauopathies have been described [36]. Although the 3R/4R isoform of tau that these tracers binds to overlaps with other tauopathies, the spatial distribution of tracer binding may help discriminate between pathologies [35].

Unlike Aβ plaque deposition, human post-mortem studies indicate that NFT density correlates with neurodegeneration and cognitive impairment [37, 38]. Several tau PET studies have shown a close relationship between patterns of tau deposition and atrophy measures [39–41] and recent work has shown memory scores to be strongly correlated with medial temporal tau tracer uptake, whereas whole-brain measures showed weak associations with memory and MTL atrophy, supporting the notion that regional tau measures have greater sensitivity to early neurodegeneration and memory decline compared to global measures of tau [39, 42]. Older age is also associated with binding in the medial temporal lobe (MTL), the extent of which is associated with memory deficits [43]. Consistent with prior histopathological reports, PET detection of tau outside the MTL is associated with the presence of cortical Aβ binding even at a subthreshold level [26, 44]. Overall, tau imaging appears capable of detecting regionally specific patterns of tau deposition that follow Braak and Braak staging of NFT pathology [8, 39, 42]. However, disentangling primary age-related tauopathy (PART) and AD will be challenging since there is considerable overlap in the medial temporal lobe.

Of the available tracers, [^18^F]-1451 (or T-807) has been characterized the most extensively and has demonstrated increased uptake and signal detection in patients with prodromal AD [35].

While tau PET’s reliability is still under investigation, test-retest reliability of the tracer was recently examined in a sample of 21 subjects (including MCI and AD patients) and showed low variability within subject. Intra-class correlation of SUVR’s was above 0.92 across all regions tested, which indicates high test-retest reliability and suggests that this method can be used to detect changes in tau burden over time [45]. Despite its many advantages, [^18^F]-1451 and similar tracers appear to bind to some dense core plaques [46], melanin-containing structures [47, 48], and minimal binding to TDP-43 [47]. This has called into question the utility of these first-generation tracers to specifically bind to tau pathology. Second generation tracers, such as [^18^F] MK-6240 fare better in terms of off-target binding, but large-scale studies with these tracers are still lacking [49].

A major strength of tau PET imaging is the ability to recapitulate histology-based Braak and Braak staging of tau pathology. While longitudinal studies remain necessary to validate the approach, initial cross-sectional data suggest a medial temporal to isocortical progression [50]. Limitations of tau PET imaging are largely similar to amyloid PET imaging and include issues with harmonization across studies and tracers and choice of reference region.

## Imaging Neural Injury and Neurodegeneration

### Synaptic Integrity and Circuit Connectivity – Resting state fMRI

Functional MRI techniques are based on blood-oxygenation-level-dependent (BOLD) contrast which is associated with neural activity at the population level. Resting-state functional magnetic resonance imaging (rs-fMRI) studies examine the temporal correlation of the BOLD signal between the regions of interest (or functional connectivity) by analyzing task-independent spontaneous fluctuations in brain networks [51, 52]. An emerging systems-based model of AD considers the large-scale disruptions across the course of AD. In preclinical AD, studies have generally noted that resting state fMRI (rsfMRI) is linked to metabolic changes (indexed by PET imaging) and precedes neurodegeneration (review by [53]). Most analyses have focused on the default mode network (DMN) [54, 55] - a network that involves the medial prefrontal cortex, posterior cingulate cortex, precuneus, anterior cingulate cortex, parietal cortex, and the medial temporal lobe, including the hippocampus [56, 57]. As regions within the DMN are highly overlapping with the spatial distribution of both amyloid and tau pathology [57], resting state fMRI can offer important information on the integrity of these circuits and the degree to which their synaptic connectivity may be affected by the disease process. While some studies have found that alterations to DMN connectivity become more dramatic with disease progression, others have found dynamic changes that relate to Aß and tau-specific profiles [40, 58–63].

In addition to changes in the DMN, some studies have suggested that connectivity within the MTL is also disrupted with aging and AD. For example, Yassa et al. [64] showed an age-related decrease in connectivity between the entorhinal cortex and the dentate and CA3 regions of the hippocampus, the extent of which was correlated with memory deficits. Connectivity changes in other networks have also been reported [65]. For example, the interaction between the DMN and the salience network, which consists of anterior insula, dorsal anterior cingulate cortex, is associated with increased connectivity in amyloid-positive individuals with low neocortical tau, and decreased connectivity as a function of elevated Tau-PET signal [62]. Functional connectivity is thought to be an early marker of synaptic pathology that may be associated with isolation of the hippocampus from its cortical input.

### Reduced Inhibition and Hippocampal Hyperactivity – Task activated fMRI

Numerous studies have used task-activated fMRI to examine functional changes in MCI and early AD. Dickerson and colleagues [66] found increased hippocampal activity during learning in individuals with MCI compared to normal controls and individuals with AD. Another study [67] using an independent component analysis found that less impaired MCI patients showed this increase, while more impaired MCI patients showed a decrease in activity similar to mild AD cases [66, 68]. These results suggested that hippocampal hyperactivation was temporally constrained. Additional data from [69] showed that the extent of hippocampal hyperactivation at baseline predicted cognitive decline as measured by the CDR-SB scores over four years after scanning. High-resolution fMRI studies have shown that this hippocampal hyperactivity is specific to the dentate and CA3 subregions of the hippocampus [70]. Recent work has also shown that this effect is noted in cognitively intact *APOE* ε4 carriers [71]. Studies in aged rodents with memory deficits suggest that CA3 hyperactivity may be due, at least in part, to the loss of GABAergic drive in hilar inhibitory interneurons, particularly somatostatin-positive (SOM+) interneurons [72].

Using domain-selective tasks that differentially engage the anterolateral (aLEC) and posteromedial entorhinal cortex (pMEC), Reagh et al. [73] found an age-related imbalance in the aLEC-DG/CA3 circuit characterized by reduced signaling in the aLEC that is coupled with increased signaling in DG/CA3 in the absence of structural thinning of the regions. These findings suggest that hyperactivity in the DG/CA3 region may, in part, be due to disruptions in the aLEC-DG/CA3 circuit via degeneration of the perforant path. Recent evidence also suggests that hippocampal activation is associated with longitudinal amyloid accumulation and cognitive decline [74].

This elevation in hippocampal activity can be targeted with pharmacological manipulations such as low-dose levetiracetam (LEV; an antiepileptic), which has shown positive results in a proof-of-concept trial. The drug successfully reduced hyperactivity in the hippocampus and reduced memory deficits in patients with amnestic MCI [75]. Later work showed that this effect was limited to the lower dosage of the drug and disappeared when higher doses were used [76] suggesting an alternative mechanism at higher doses. Interestingly, LEV targets synaptic vesicle protein SV2A which can now be imaged using a novel PET tracer (see last section on new approaches). Additionally, low dose LEV fully restores hilar SOM expression in aged, memory-impaired rats [72], suggesting that restoring inhibition may be a critical therapeutic path and that high-resolution functional MRI may be a suitable method to assess target engagement and therapeutic efficacy in clinical trials.

### Reduced white matter integrity – Diffusion MRI

Diffusion tensor imaging (DTI) has been used to investigate the microstructural features of white matter [77]. The majority of DTI studies assess white matter integrity using voxel-wise values such as fractional anisotropy (FA), which is a scalar quantity that measures the anisotropy (i.e. directionality) of the diffusion signal in any given voxel. There are many factors that affect FA including axonal degeneration, demyelination, disorganization, packing density, and other microstructural features, but it is often measured as an indirect proxy to white matter integrity. Although the neural basis of anisotropy is still not completely understood, it has been used as an index of white matter integrity in thousands of studies across humans and animals. Typically the higher the FA value, the more intact a fiber pathway is thought to be.

A number of DTI studies have shown white matter loss with aging (see review by Chua et al. [78]), most likely due to thin myelinated fiber degeneration [79–82]. DTI studies of MCI and AD show widespread declines in white matter integrity throughout the brain with the most reliable changes reported in the temporal lobes [78, 83–86].

Investigations of white matter connectivity changes in aging and AD have focused on the fornix and the cingulum, as they are the major links between the limbic system and the rest of the brain. The fornix is the largest input/output fiber bundle of the hippocampus and connects it to the hypothalamus, while the cingulum connects the cingulate and the parahippocampal gyri to the septal cortex. Damage to the fornix has been found to reproduce learning and memory deficits resulting from hippocampal lesions in rats [87, 88] and in monkeys [89–91]. DTI fiber tracking studies show reduced fractional anisotropy in the fornix in AD [92, 93]. Several studies have found white matter changes in the cingulum in MCI and mild AD cases [94–96].

The perforant path connects EC layer II neurons to the hippocampal DG and CA3 [97] and is critical for normal hippocampal function [98]. This pathway’s integrity is reduced in aged rats with memory loss [99, 100]. Perforant path lesions also result in EC layer II neuronal loss [101], one of the earliest hallmark features of AD. Thus, attempts to evaluate perforant path alterations are critical to understanding early AD pathophysiology.

Numerous studies have shown changes in parahippocampal white matter in aging and MCI using structural MRI and diffusion tensor imaging (DTI) [102–105]. However, since there are many crossing fibers in the region and the perforant path is only ~ 2-3 mm thick fiber sheet, it was not possible to uniquely ascribe these changes to the perforant path itself. More recent work used an ultrahigh resolution (submillimeter) DTI technique to assess the perforant path [106, 107], which was validated against post-mortem data [108]. This method more specifically allowed for imaging the perforant path and documented loss of integrity with aging in a manner that was related to the extent of memory deficits.

Traditional DTI approaches are limited by the inability to resolve intra-voxel complexities such as fiber bending, crossing, and twisting [109]. High angular resolution diffusion imaging (HARDI) addresses this limitation by sampling the diffusion signal along many more gradient directions and providing adequate information to model diffusion with an orientation distribution function (ODF), a more versatile diffusion representation that captures multiple orientations in a voxel [110]. Given the complexity of white matter and the specific patterns of atrophy related to AD, HARDI may offer an improved approach to biomarker discovery.

### Cortical thinning and volume loss – Structural MRI

Compared with images from other modalities, MR images provide excellent anatomical detail and additionally provide a strong grey/white matter contrast. Processes believed to be pathological in nature are often described in terms of anatomical location, cortical thickness, volumetry, and morphological characteristics.

Coronal T1-weighted, three dimensional, high resolution images are often used in cross-sectional and longitudinal studies to measure the hippocampal volume and to assess changes in hippocampal volume over time in AD [111, 112]. They have also been used to reveal many age-related changes in the brain. There is a decrease in total brain volume resultant from cortical thinning and gyral atrophy [113]. Specifically, the prefrontal cortex and the hippocampal formation display volume loss in advanced aging that significantly accelerates from normal aging to MCI to AD [114, 115].

Volume and shape changes in the hippocampus have been shown with healthy aging and preclinical AD [115–118]. Some MRI studies have also shown that the extent of hippocampal and entorhinal volume decline with increasing age predicted performance on memory tasks [119, 120]. Despite these studies, it is not clear whether any of these changes are actually the result of frank cell loss with age, or perhaps are secondary to synaptic and dendritic loss. Studies in aged rodents and non-human primates have reliably demonstrated the absence of frank cell loss in the hippocampus with age [121–123], but regions in the prefrontal cortex are found to undergo cell loss [124–126].

Although dramatic neuronal loss is not observed in preclinical AD or MCI, several studies have shown mild hippocampal atrophy during these stages. Hippocampal atrophy has been linked to cognitive impairment suggestive of AD [127–129]. Several human structural MRI studies have used very-high-dimension transformation techniques to observe changes in the shape of the hippocampus associated with AD. Consistent with the histological data, changes in the area of the CA1 fields in the hippocampus have been reported [130, 131]. Notably, in one of these studies, the same region of CA1 identified as differing in shape between non-demented and mildly demented patients also varied in the non-demented patients as a function of whether or not they later converted to a CDR (Clinical Dementia Rating) of 0.5 [130]. More recent work by the same group suggests that surface deflections across all hippocampal subfields (CA1 lateral zone, dentate gyrus/CA2-4 superior zone, and subiculum inferior medial zone) differentiate non-demented controls from early AD patients [132].

Recent high-resolution structural imaging studies in MCI patients where subfields of the hippocampus were manually segmented have suggested that specific subfields are more vulnerable than others. Yassa et al. [70] found that the CA1 and CA3/dentate gyrus regions both show volumetric loss, with left-lateralized changes in both subregions. The subiculum and other medial temporal regions were no different in MCI patients and controls. Similar techniques showed that the subiculum, CA1, and entorhinal cortex are further affected in AD [133, 134]. Mueller and Weiner [133] also found that *APOE* ε4 status was associated with volumetric decline in the CA3/dentate subregions, suggesting that early risk for AD may selectively affect this region, and is consistent with the loss of synaptic input reported in animal studies.

Subfield-specific patterns of atrophy are complex and require improved segmentation of hippocampal subfields that are both reliable and histologically validated. Current efforts by the Hippocampal Subfield Group (HSG: http://hippocampalsubfields.com) is making advances in this direction [135, 136]. Higher resolution scans at increased MRI strength (7T) have also shown promise in examining changes in particular layers of the hippocampal region that may be vulnerable at early stages of the disease. The apical dendrites of hippocampal CA1 pyramidal neurons, in the stratum radiatum/stratum lacunosum-moleculare (SRLM), are targeted by tau pathology early in the course of disease. Several studies have shown that using high-resolution (~200 micron) T2-weighted scans at 7T allows for identification and assessment of SRLM and demonstrate AD-related atrophy [137]. Similar changes have also been noted in nondemented older adults [138, 139] and in *APOE* ε4 carriers [140].

In recent years, cortical thinning in the entorhinal cortex (EC) has been identified as a highly sensitive measure of structural change both in MCI and AD [141]. EC thickness diminishes prior to, and predicts, hippocampal atrophy [142–145]. Several recent studies using the Alzheimer’s Disease Neuroimaging Initiative (ADNI) data have shown evidence of EC thinning in older adults with CSF pathological markers of AD (Aβ and p-Tau) [144, 146]. Another recent study by Ewers et al. [147] suggested that EC loss was one of the best predictors of MCI conversion to AD, even surpassing multimarker models.

Thus, results from structural MRI studies have generally shown that both the entorhinal cortex and the hippocampus show robust volumetric declines in MCI and AD (with the entorhinal change occurring earlier) and may be used as an early diagnostic feature. Limitations of the methods include differences in spatial resolution across scans, susceptibility to movement, and difficulties in determining the neural source of volume or thickness loss (cell loss vs. dendritic and synaptic loss) without exceptionally high-resolution scanning that is not feasible for most institutions.

### Cerebral glucose hypometabolism – FDG-PET

PET methods have been used for over three decades to examine alterations in brain glucose metabolism in aging, MCI and AD [148]. Regional cerebral metabolism can be assessed with ^18^F-2fluoro-2-deoxy-D-glucose (FDG) as a metabolic marker. In particular, findings of reduced hippocampal metabolism in MCI and AD have been reported [149]. Cerebral glucose hypometabolism on FDG-PET appears to be a downstream marker of neuronal injury and neurodegeneration. In particular, it appears reliably in temporal, parietal (and possibly frontal) lobes but spares sensorimotor cortices, visual cortices, basal ganglia, thalamic nuclei and the cerebellum [150].

Importantly, age-related patterns of cerebral glucose metabolism differ substantially from patterns observed in AD, which has led to the utility of this technique in aiding clinical diagnosis. While classic studies (e.g. [151]) have shown that average cerebral glucose metabolism decreases with age, the regions showing the least age-related change include the medial temporal lobes, the posterior cingulate cortex and the precuneus. Those are the same regions expressing significant hypometabolism in AD. Thus, FDG-PET can be used to determine if the pattern of cerebral hypometabolism is normal or abnormal. Mosconi et al. [152] showed that it can be used to differentiate AD patients from healthy subjects with 99% sensitivity and 98% specificity.

Studies have also suggested that FDG-PET can be quite accurate at differentially diagnosing AD from other dementias and has a high concordance rate with clinical diagnosis [153]. That said, recent results have also suggested that hypometabolism in one of the key regions implicated in AD, the posterior cingulate cortex, cannot be used in isolation for differential diagnosis, as a subset of patients with the behavioral variant of frontotemporal dementia also show this pattern of hypometabolism [154].

While it has been suggested that structural MRI and FDG-PET can be used interchangeably to index neurodegenerative processes, more recent data suggest that they offer complementary and non-overlapping information. For example, Benvenutto et al. [155] showed that the extent of glucose hypometabolism can be used to track clinical severity, whereas structural MRI markers had higher associations with higher educational attainment (higher cognitive reserve). Other work has also shown that FDG-PET can be used to predict conversion from MCI to AD (odds ratio of 84.9%) [156].

Recent work by the Alzheimer’s Disease Neuroimaging Initiative (ADNI) 2 PET Core have examined the combined utility of FDG-PET and amyloid PET at tracking progression of the disease. For example, they demonstrate that amyloid PET (using florbetapir uptake) is negatively associated with temporoparietal metabolism [157]. In healthy controls, florbetapir was associated with cognitive change, whereas in MCI patients FDG-PET metabolism was associated with cognitive change [158]. This is consistent with the biomarker model in which amyloid aggregation precedes neurodegeneration.

Limitations of FDG-PET include all of the limitations previously discussed for other PET-based approaches including harmonization of procedures and analyses. However, given the long history of FDG-PET scanning, these methods are far more standardized than amyloid or tau imaging.

## Emerging Methods

The final section discusses some of the most exciting emerging methods that may potentially allow us to add new and informative biomarkers to the AT(N) criteria. In addition to protein aggregation and cellular injury/neurodegeneration, AD is characterized by increased inflammation, epigenetic dysfunction, and synaptic loss. The three emerging methods we discuss below attempt to break new ground in imaging and tracking these pathologies in vivo.

### Imaging Neuroinflammation (TSPO-PET)

Translocator protein (TSPO) is an outer mitochondrial membrane protein that is expressed in many tissues throughout the body [159]. In the healthy brain, TSPO is only expressed at low levels and its expression is upregulated in activated and proliferating microglia and astrocytes following brain injury and neuroinflammation [160–162]. The differential expression of TSPO in activated glia enables for it to be exploited with PET to observe and quantify neuroinflammatory changes. Thus, PET tracers for TSPO were developed over the past two decades as markers for glial activation and neuroinflammation in AD. The attempts have had mixed results.

The first PET study with a TSPO tracer in AD patients was published by Cagnin et al. [163] and showed an increased uptake of the [^11^C]-based tracer PK11195. Later reports provided mixed results with some studies showing weak links between microglial activation and AD progression [164] and a poorly understood relationship with amyloid beta deposition [165, 166]. It became clear that TSPO tracers had limitations including a modest binding affinity, high non-specific binding, and low signal-to-noise ratio [167].

Second generation tracers were subsequently developed to improve these limitations. However, they were affected by genetic variability of the TSPO binding site due to the rs6971 single-nucleotide polymorphism, which resulted in high-affinity, mixed-affinity, and low-affinity binders [168]. This effectively limited the use of the tracer to studies only in high and mixed-affinity binders, and required a genetic test prior to the scan. One recent study with the [^11^C]-PBR28 PET tracer has also found significant widespread clusters positively correlated between levels of microglial activation and tau aggregation via [^18^F]-AV1451 PET imaging in MCI and AD subjects [169]. The correlations were stronger in AD than MCI. However, levels of microglial activation and amyloid deposition were also correlated, and the correlations were stronger in MCI than AD. This would suggest that microglial activation can correlate with both tau aggregation and amyloid deposition.

Third generation tracers, such as GE-180 were produced with the intent of TSPO quantification regardless of genotype, [170, 171]. Early data suggests that increased TSPO binding is associated with various dementias, but more studies are needed in AD patients. Although, TSPO imaging may potentially serve as a biomarker for neuroinflammation, future development of these tracers and enhancing their specificity and sensitivity will be needed [172].

### Imaging Epigenetics (^11^C-Martinostat PET)

Epigenetics refers to a set of molecular mechanisms that are involved in regulating gene expression, but which do not involve alterations to the genetic code itself. They include modifications to the structure of the DNA (methylation) or modifications of the chromatin (acetylation). Chromatin includes DNA and the histone proteins that help package genomic DNA into the nucleus of a cell. Epigenetic modifications are thought to be involved in the dynamic process of learning and memory and are altered by aging and AD pathology.

Whether epigenetic alterations contribute causally to AD or are a consequence of upstream events still remains subject to debate [173]. Certain epigenetic changes may arise before AD pathology presents [174] and some may be more downstream [175, 176]. In both cases, understanding the changes to the epigenetic landscape that occur prior to, and during, the progression of AD can significantly enrich our understanding of disease pathophysiology.

Histone acetylation is a particular type of epigenetic modification controlled by histone acetyltransferases (HATs), which add acetyl groups to histone proteins, and histone deacetylase (HDACs), which remove acetyl groups from histone proteins. Imaging this process in vivo in humans would allow for a means to assess the epigenetic landscape. The novel radiotracer [^11^C] Martinostat allows for imaging HDAC density with high specific binding of a subset of class I HDAC enzymes (isoforms 1, 2, and 3), favorable kinetics, and high affinity [177]. In human studies, HDAC expression was higher in cortical gray matter than white matter and was generally lowest in the amygdala and hippocampus [178]. Follow-up work by the same group developed a fluorinated variant of the tracer [^18^F] MGS3 [179], which exhibits specific binding, comparable brain uptake and regional distribution to [^11^C] Martinostat, however the radiosynthesis process remains highly inefficient precluding complete validation using blocking experiments in nonhuman primates and subsequent use in humans. Epigenetic imaging may soon offer a unique look into gene regulatory processes that are implicated in AD, however, it is still too early at this time to determine its utility as a biomarker for AD.

### Imaging Synapses (^11^C-UCB-J PET)

Synapse loss is an important feature of neurodegeneration, and it precedes cellular degeneration in most cases. Observing synaptic loss in humans has not been possible until recently with the advent of novel PET tracers for synaptic vesicle proteins. The synaptic vesicle protein 2A (SV2A) found in neurons as well as endocrine cells is essential for synaptic neurotransmitter release and is targeted by anti-epileptics such as levetiracetam. Thus, it can potentially serve as a biomarker for synaptic density. The recent development of the SV2A PET radiotracer [^11^C] UCB-J [180] may offer the possibility of imaging synaptic density in vivo, and potentially inform biomarker science not just for AD but for numerous other conditions involving synapse loss [181].

A recent study by Chen et al. [182] used [^11^C] UCB-J to quantify SV2A binding in a small sample of AD patients (amyloid positive) and healthy controls (amyloid negative). The authors found a significant reduction in SV2A binding in AD patients compared to healthy controls in addition to a relationship between overall SV2A binding and episodic memory scores. For decades, the only information that could be gleaned about synaptic integrity was indirectly through FDG-PET scans which are thought to be an indirect correlate of synapse loss given the relationship between glucose metabolism and synaptic markers. However, with this new advance, the field has the opportunity to directly examine synapses [183]. While still in the early stages, this work ushers promise in understanding the nature of synaptic alterations in AD and a host of other neurological illnesses.

## Summary and Conclusions

In vivo neuroimaging in humans provides a richer understanding of the pathophysiology of AD. We discussed a number of methods that have already provided useful information in terms of diagnosing the disease during the preclinical stage, tracking its progression, and testing the efficacy of disease modifying therapeutics. For these methods to allow us to develop appropriate biomarkers that can serve as meaningful outcomes or surrogate endpoints they have to meet numerous criteria.

At a minimum, we suggest that across all these biomarkers, investigators should think carefully about test-retest reliability (see review by Henriques et al.[184]), histological validation, specificity to the disease process, sensitivity to detect abnormalities when they are subtle, practical feasibility in a clinical research setting, and relationship to cognitive/clinical outcomes. At this time, there is not a single imaging modality that meets all of the above criteria and singularly provides a rich enough understanding of pathological processes. Not only do different imaging modalities offer complementary information, but the spatial distribution of the measurements can also offer rich information that can be used for tracking and staging within individuals and groups.

Thus, we suggest that there is a need for composite neuroimaging biomarkers that combine information about glial inflammation, epigenomic alterations, amyloid and tau aggregation, structural and functional alterations, and synaptic and cellular degeneration. With the growing number of large-scale multimodal datasets (e.g. ADNI), there is a growing need for developing precision medicine approaches to better characterize, stage, and classify subtypes of dementias and discriminate AD from age-related changes.

Enabling precision medicine research in AD was identified as a key recommendation resulting from the National Institute on Aging (NIA)’s Alzheimer’s Disease Research Summit 2018: Path to Treatment and Prevention. The use of robust artificial intelligence and the integration of neuroimaging data with other -omics data will be critical to advance in the field of Alzheimer’s disease therapeutics.

**Table:** 

Modality	Major Finding	References
Amyloid-PET	Amyloid deposition is linked to aberrant entorhinal activity among cognitively normal older adults	[22]
	Subthreshold amyloid deposition predicts tau deposition in aging	[26]
	Increased Aβ is associated with cortical thinning in frontoparietal regions	[24]
Tau-PET	Tau deposition predicts atrophy measures	[39–41]
	Higher tracer uptake in the parahippocampal gyrus strongly relates to episodic memory	[185]
	Older age is associated with binding in the medial temporal lobe (MTL), the extent of which is associated with memory deficits	[43]
	Memory scores are strongly correlated with medial temporal tau tracer uptake, whereas whole-brain measures showed weak associations with memory and MTL atrophy	[42]
Task-Activated fMRI	Increased hippocampal activity during learning in individuals with MCI compared to normal controls and individuals with AD.	[66]
	Less impaired MCI patients showed this increase, while more impaired MCI patients showed a decrease in activity similar to mild AD cases	[67]
	More impaired MCI patients showed a decrease in activity similar to mild AD cases	[68]
	The extent of hippocampal hyperactivation at baseline predicted cognitive decline as measured by the CDR-SB scores over four years after scanning.	[69]
	High-resolution fMRI studies have shown that this hippocampal hyperactivity is specific to the DG/CA3 subregions of the hippocampus	[70, 71]
	Reduced signaling in the LEC coupled with increased signaling in DG/CA3 in the absence of structural thinning of the regions.	[73]
	Hippocampal activation is associated with longitudinal amyloid accumulation and cognitive decline	[74]
Resting-State fMRI	Widespread changes in DMN connectivity in MCI and AD	[58–61]
	Hyperconnectivity in the anterior DMN and hypoconnectivity in the posterior DMN in AD	[63, 186]
	Aß+ and tau-PET signal specific profiles	[62, 187]
	Age-related decrease in connectivity between the entorhinal cortex and the dentate and CA3 regions of the hippocampus, the extent of which was correlated with memory deficits.	[64]
Diffusion MRI	Widespread changes in white matter in MCI and AD	[83–86]
	DTI fiber tracking studies show white matter microstructural changes in the fornix and cingulum in MCI and mild AD cases	[92, 94–96, 107]
	Parahippocampal white matter changes in aging and MCI using structural MRI and diffusion tensor imaging (DTI)	[93, 102–105]
	Perforant path degradation in non-demented older adults	[106–108]
Structural MRI	Volume and shape changes in the hippocampus with healthy aging and preclinical AD	[115–118]
	Volumetric loss of CA1 and DG/CA3 in APOE4 carriers, preclinical AD, MCI and AD in high resolution scans	[70, 130–132]
	ERC thickness predicts hippocampal atrophy (including CA1-SRLM size) and is a sensitive measure of structural change in MCI and AD	[133, 139, 141–143, 147]
TSPO-PET	In the healthy brain, TSPO is only expressed at low levels and its expression is upregulated in activated and proliferating microglia and astrocytes following brain injury and neuroinflammation	[160–162]
	AD patients show an increased global and regional uptake	[163, 165, 166]
	Microglial activation can correlate with both tau aggregation and amyloid deposition.	[169]
Epigenetic modifications	In healthy adults, HDAC expression was lowest in the hippocampus and amygdala among gray matter regions	[178]
Imaging Synapses	Significant reduction in SV2A binding in AD patients compared to healthy controls in addition to a relationship between overall SV2A binding and episodic memory scores.	[182]

## Data Availability

Not applicable
